# Effects of carbon monoxide, nitrogen dioxide, and fine particulate matter on insect abundance and diversity in urban green spaces

**DOI:** 10.1038/s41598-022-20577-x

**Published:** 2022-10-25

**Authors:** Minoo Heidari Latibari, Gholamhossein Moravvej, Diana Carolina Arias-Penna, Mostafa Ghafouri Moghaddam

**Affiliations:** 1 Department of Plant Protection, College of Agriculture, Ferdowsi University of Mashhad, P. O. Box: 91779–48974, Mashhad, Iran; 2 Unaffiliated Entomologist, Bogotá, Cundinamarca Colombia; 3 Integrative Ecology Laboratory, Department of Biology, Faculty of Science, Chulalongkorn University, Phayathai Road, Pathumwan, Bangkok, 10330 Thailand

**Keywords:** Zoology, Environmental sciences

## Abstract

The impact of three air pollutants, carbon monoxide, nitrogen dioxide, and fine particulate matter (PM_2.5_) on the diversity and abundance of insect fauna associated with Chinese thuja, *Platycladus orientalis*, was evaluated for the first time in Iran. Sampling was performed in Lashkar, Sadaf, and Sajjad, three urban green spaces in Mashhad, a city located in Razavi Khorasan province. A total of 29 insect species including 16 natural enemies and 13 herbivores were identified. The results indicated that herbivore abundance was positively and significantly correlated with the level of carbon monoxide and nitrogen dioxide, but not with the level of fine particulate matter. In contrast, herbivore diversity was negatively and significantly correlated only with the level of carbon monoxide. As for natural enemies, abundance and diversity both were not significantly correlated with the levels of none of all three pollutants. The aphid, *Cinara thujafilina* Del Guercio was the most dominant herbivore species in all the sampling sites with a relative abundance of 86% at Lashkar, 93% at Sadaf, and 95% at Sajjad. Regarding natural enemies, the ladybird, *Coccinella septempunctata* was the most abundant species both at Lashkar (49%) and Sadaf (35%) while the ladybird, *Oenopia conglubata* was the most abundant at Sajjad (21%). The highest diversity of herbivores and natural enemies across the four seasons were at the Lashkar and Sadaf sites. The results highlight that of the three air pollutants, carbon monoxide is the one that affects both on abundance and diversity of herbivore guild associated with *Platycladus orientalis* in urban green spaces.

## Introduction

Air is one of the five main elements for the continuation of human life, but air pollution has become a serious challenge in most countries. Pollutants are divided into two main categories, natural pollutants such as sandstorms, volcanic gas emissions, pollen from plants, etc., and artificial pollutants which include the gray soot that cars, factories, and power plants produce^1^. According to the United States, Environmental Protection Agency (EPA), any gaseous, liquid, solid, radionuclide, or non-radionuclide radiation or anys combination of soot, suspended particles, sulfur oxides (SO_x_), nitrogen oxides (NO_x_), carbon monoxide (CO), oxidizers, hydrocarbons, acids, and ammonia (NH_3_) that are released into the air are considered as polluted air^1^.

The ongoing development of cities worldwide has changed dramatically over the last 100 years and cities are expanding significantly faster than 50 years ago. The growth of the cities has had a considerable impact on local hydrology, land cover, and surrounding environments through the building materials used in the cities^2^. A majority of humans now reside in cities and that number will continue to increase just like cars, factories, and power plants. The primary air pollutants are ozone (O_3)_, particulate matter (PM_10_ and PM_2.5._), carbon dioxide (CO_2_), CO, sulfur trioxide (SO_3_), nitrogen dioxide (NO_2_), and nitrogen monoxide (NO). The air quality in different cities and countries across the world is declining and since the cities are the main contributor to air pollution on local, national, and international scales, it is important to recognize the contaminating agents of air pollution and its destructive effects on the environment. Such investigations are essential elements for the improvement and preservation of the city’s environmental quality^1^. Carbon monoxide, NOx, SOx, and solid particles (dust) are the most common air pollutants that come from anthropogenic sources. These pollutants are some of the primary causes of acute and chronic respiratory diseases, skin cancer, and exacerbation of heart disease in humans likewise deteriorating the surfaces of buildings and monuments^3^. Air pollutants also have an adverse effect on the performance of insect natural enemies, as they reduce their ability to locate hosts^4–6^. This is concerning since the use of biological control agents is an important strategy to help regulate populations of insect pests^6^. Therefore, it is essential to study how the diversity of natural enemies is impacted by air pollution.

Primary (direct) and secondary effects (indirect) of some air pollutants such as fluorine, lead, sulfur, ozone, and dust particles have been studied on insect populations. Results revealed that air pollutants directly reduce the abundance of economically useful insect populations such as silkworms (*Bombyx mori* (L.), Lepidoptera: Bombycidae), and honeybees (*Apis mellifera* L., Hymenoptera: Apidae). Secondary effects of air pollution results in the increase of pest insects, such as bark beetles (Coleoptera: Scolytidae) and aphids (Hemiptera: Aphididae), which decrease plant resistance against herbivores^4^. Several studies have been conducted on the effects of air pollution on plants and other living organisms^7^ and showed that it can disrupt the interaction between plants, herbivores, and their natural enemies^8^.

Air pollution is one of the most important shared challenges among megacities or metropolises across the world, especially in developing countries such as Iran. Today, many important cities in Iran have the problem of inappropriate weather conditions, and increasing population, industries and vehicles have led to a decrease in air quality index^9^. Air Quality Index (AQI) in large cities of Iran such as Tehran, Mashhad, and Ahwaz, for more than 150 days a year is higher than the permissible standard by the Environment Department^10^. Mashhad observes more than 3.1 million cars daily^11^ which have a decisive role in air pollution along with the industries located in the city. The enormous population of inhabitants and tourists and its development pattern have raised inner-city trips, so the number of these trips has increased from 4,035,560 trips per day in 2006 to 6,241,830 trips in 2019 (annual growth of 3.4%). Most of these trips take place along 10 highways in the city, especially northwest to southeast and west to east. During this period, the consumption of petrol and gasoline reached from 179,565 to 2,353,237 liters (annual growth of 21.8%), leading to an increase in air pollutants^11^. It should be noted that the multiplicity of motor vehicles is only one of the most important causes of air pollution in Mashhad. At the same time, high population, polluting industrial centers, special climatic conditions, and being located between two mountains (Binalood and Hezar Masjed) can be effective^12^. In the case of industries, currently, about 6500 industrial units are located in and around Mashhad with an area of 86,000 ha. As a result of the physical expansion of the city in recent decades, most of these industries are located within the city^12^. Most industrial units use fuel oil and diesel which are very effective in increasing air pollution^13^. Mashhad ranks 617th in air pollution in the world and second in the country^12,14^.

A gradual increase in air pollution in Mashhad city has been reported over a long period and no data are available on its effects on the insect fauna, except the reported here. One of the actions taken by the Mashhadi authorities is to expand open green spaces within the city. Ornamental shrubs such as conifers besides providing a valuable decorative element in urban green spaces contribute to the accumulation of a considerable proportion of air pollutants, even in winter. This is the case of *Platycladus orientalis* (L.), (Pinales: Cupressaceae), known as Chinese thuja, a plant native to northeastern parts of East Asia and North Asia and introduced in other regions of the Asian continent. Chinese thuja is an important urban tree, which absorbs air pollutants, but shows high resistance to them and also helps to reduce air temperatures which are especially important due to the urban heat island effects^15^.

In this paper, insects associated with *Platycladus orientalis*, a dominant tree species in the tree-lined avenues of the city of Mashhad, Iran, were used to evaluate the effects of three air pollutants (carbon monoxide, nitrogen dioxide, and particulate matter PM_2.5_) on the diversity and abundance of both herbivores and natural enemies. For this purpose, air pollutants concentrations in three urban green spaces collected by the Environmental Pollutants Monitoring Center in Mashhad were used. Finally, the standard levels of seven air pollutants reported by Australia, the European Union, the United States, and the World Health Organization were compared to see how close or how far they are from the allowable air pollutant emissions in Mashhad.

## Results

**Figure 1:**
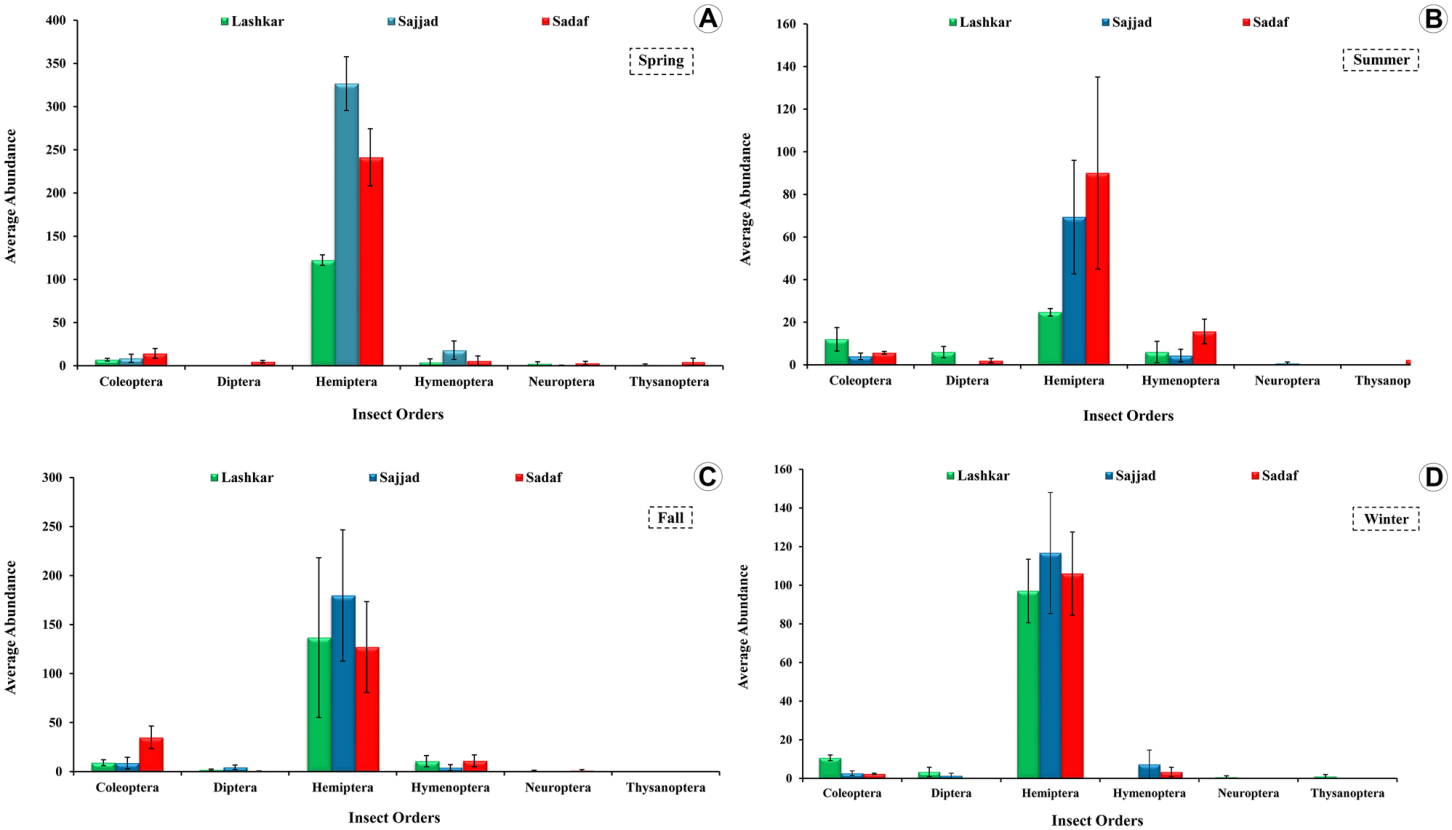
Species abundance of insect orders associated with the Chinese thuja trees, *Platycladus orientalis* (L.) during the four seasons in three urban green spaces in Mashhad, Iran. (**A**) Spring. (**B**) Summer. (**C**) Fall. (**D**) Winter.

**Figure 2:**
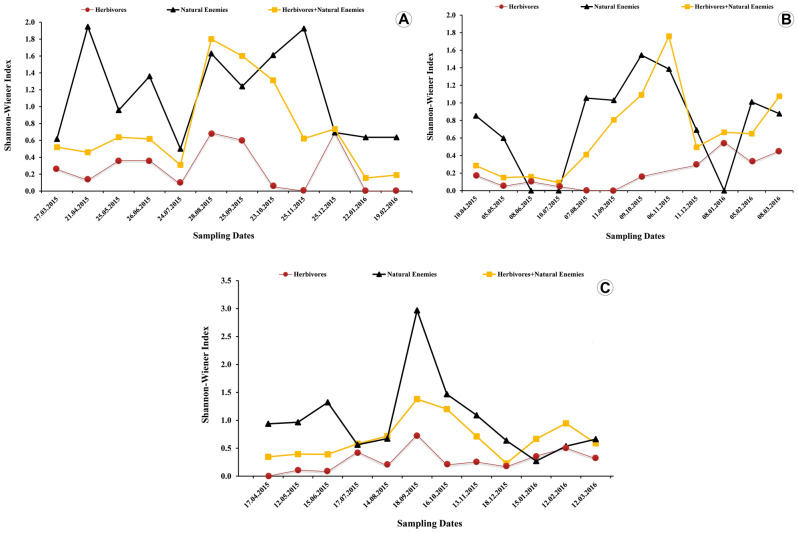
Time graph showing temporal variation in Shannon–Wiener index. Insect diversity associated with *Platycladus orientalis* (L.) in (**A**) Sadaf, (**B**) Sajjad, and (**C**) Lashkar, three urban green spaces in Mashhad city, Iran.

**Figure 3:**
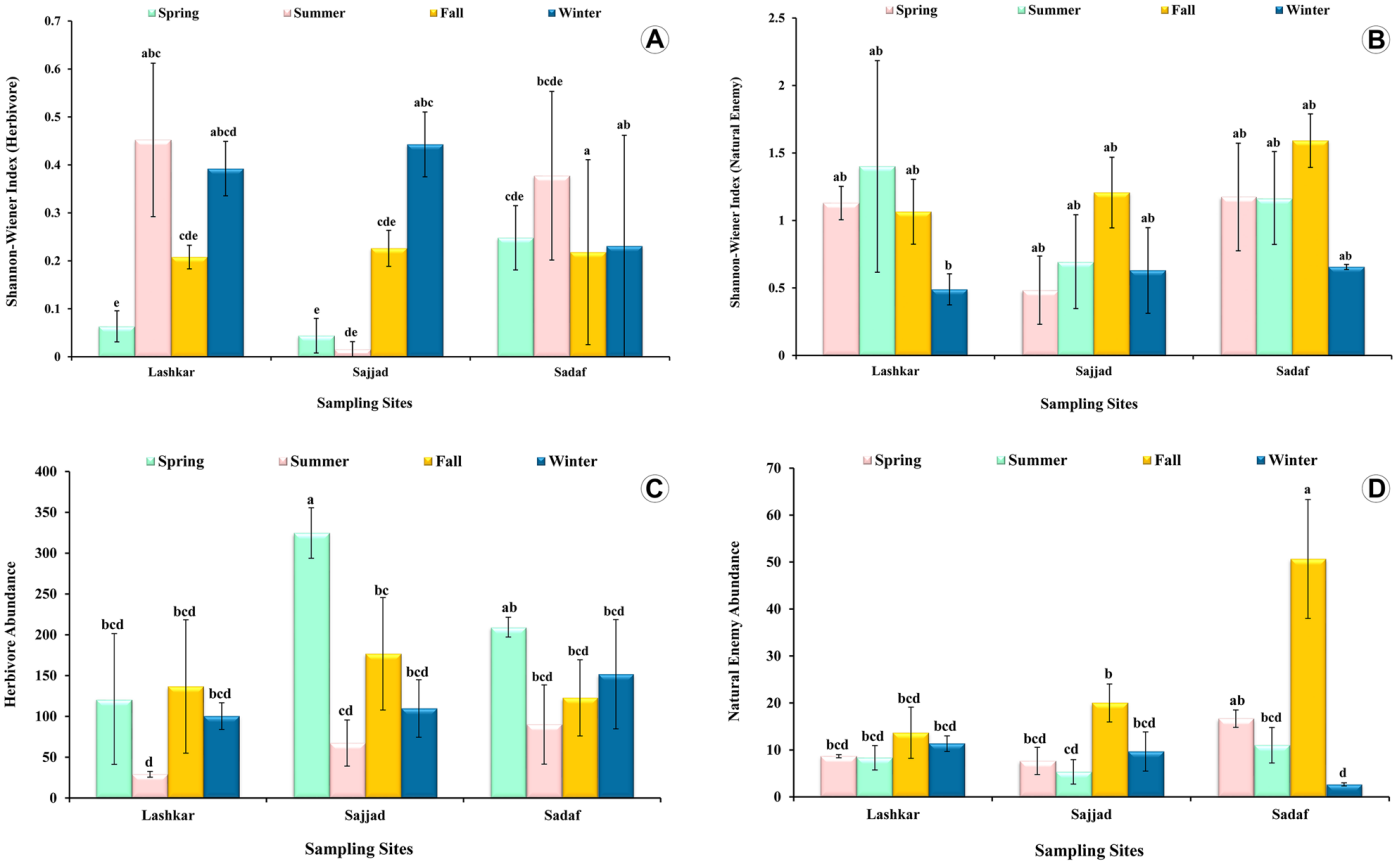
The mean Shannon–Wiener index for diversity and the mean abundance of (**A, C**) Herbivores and (**B, D**) natural enemy associated to Chinese thuja trees, *Platycladus orientalis* (L.) in three urban green spaces of Mashhad city from Iran during the four seasons. Values of zero are removed from the analysis, ± standard error, n = 3, similar lowercase letters (e.g., ab, bc, bcd, abcd, bcde, etc.) indicate there is no statistically significant difference among different seasons of each sampling site.

A total of 5404 insect specimens were collected and 29 species were identified which included 13 herbivores and 16 natural enemies (Table 1). Five orders were reported, Coleoptera (13 spp.), Hemiptera (10), Diptera, Hymenoptera, and Thysanoptera (each one with 2). Hemiptera was the most common order collected in all three sampling sites during the four seasons (Fig. 1A–D). The overall diversity (H + P), as well as the diversity of herbivores and natural enemies at each sampling site, varied significantly across different dates (Fig. 2A–C). The highest agglomeration of insects (H + P) occurred during the springtime (May 2015) in the Sajjad site and the lowest was reached during the summer (July 2015) in the Lashkar site (Fig. S1). The maximum number of herbivores was collected at the Sajjad site during spring (Fig. 3C) whereas the maximum number of natural enemies was reported at Sadaf sites during fall (Fig. 3D).

**Table 1:** Insect species associated with the Chinese thuja trees, *Platycladus orientalis* (L.) in Lashkar (**La**), Sadaf (**Sf**), and Sajjad (**Sj**), urban green spaces in Mashhad, Iran.

Order	Family	Species	Abundance		
			La	Sj	Sf
Coleoptera	Coccinellidae^p^	*Chilocorus bipustulatus* Linnaeus, 1758	2	–	1
		*Clitostethus arcuatus* Rossi, 1794	4	–	29
		*Coccinella septempunctata* Linnaeus, 1758	62	10	86
		*Coccinella undesempunctata* Linnaeus, 1758	1	8	8
		*Exochomus nigromaculatus* Goeze, 1777	1	6	11
		*Hippodamia variegata* Goeze, 1777	2	2	2
		*Oenopia conglobata* Linnaeus, 1758	17	28	18
		*Oenopia oncina* Olivier, 1808	4	1	1
		*Scymnus nubilus* Mulsant 1850	–	1	–
		*Scymnus syriacus* Marseul,1868	4	–	2
	Curculionidae^H^	*Aspidapion radiolus* Marsham 1802	–	2	–
		*Malvapion malvae* Fabricius, 1775	2	6	8
		*Smicronyx syriacus* Faust, 1887	3	9	5
Diptera	Sciaridae^H^	*Lycoriella sativae* Johannsen, 1912	19	2	13
		*Scatopsciara atomaria* Zetterstedt, 1851	6	7	10
Hemiptera	Cicadellidae^H^	*Asymmetrasca decedens* Paoli, 1932	–	19	–
		*Zyginella pulchra* Löw, 1885	32	35	138
	Aphididae^H^	*Cinara tujafilina* Del Guercio, 1909	1092	1942	1487
	Anthocoridae^P^	*Orius albidipennis* Reuter, 1884	–	8	24
		*Orius niger* Wolff, 1811	–	13	–
	Geocoridae^P^	*Geocoris luridus* Fieber, 1844	–	14	12
		*Geocoris megacephalus* Rossi, 1790	–	9	2
	Miridae^H^	*Deraeocoris lutescens* Schilling, 1837	–	18	6
		*Deraeocoris punctulatus* Fallen, 1807	–	14	7
	Aleyrodidae^H^	*Aleyrodes* sp.	–	13	40
Hymenoptera	Aphelinidae^P^	*Encarsia formosa* Gahan, 1924	–	5	34
	Braconidae^P^	*Pauesia hazratbalensis* Bhagat, 1981	2	–	3
Thysanoptera	Phlaeothripidae^H^	*Haplothrips tritici* Kurdjumov, 1912	8	–	7
		*Haplothrips subtilissimus* Haliday, 1852	–	–	17

Abundance and guilds are provided.

^H^herbivores, ^P^natural enemies that include predators + parasitoids.

**Figure 4:**
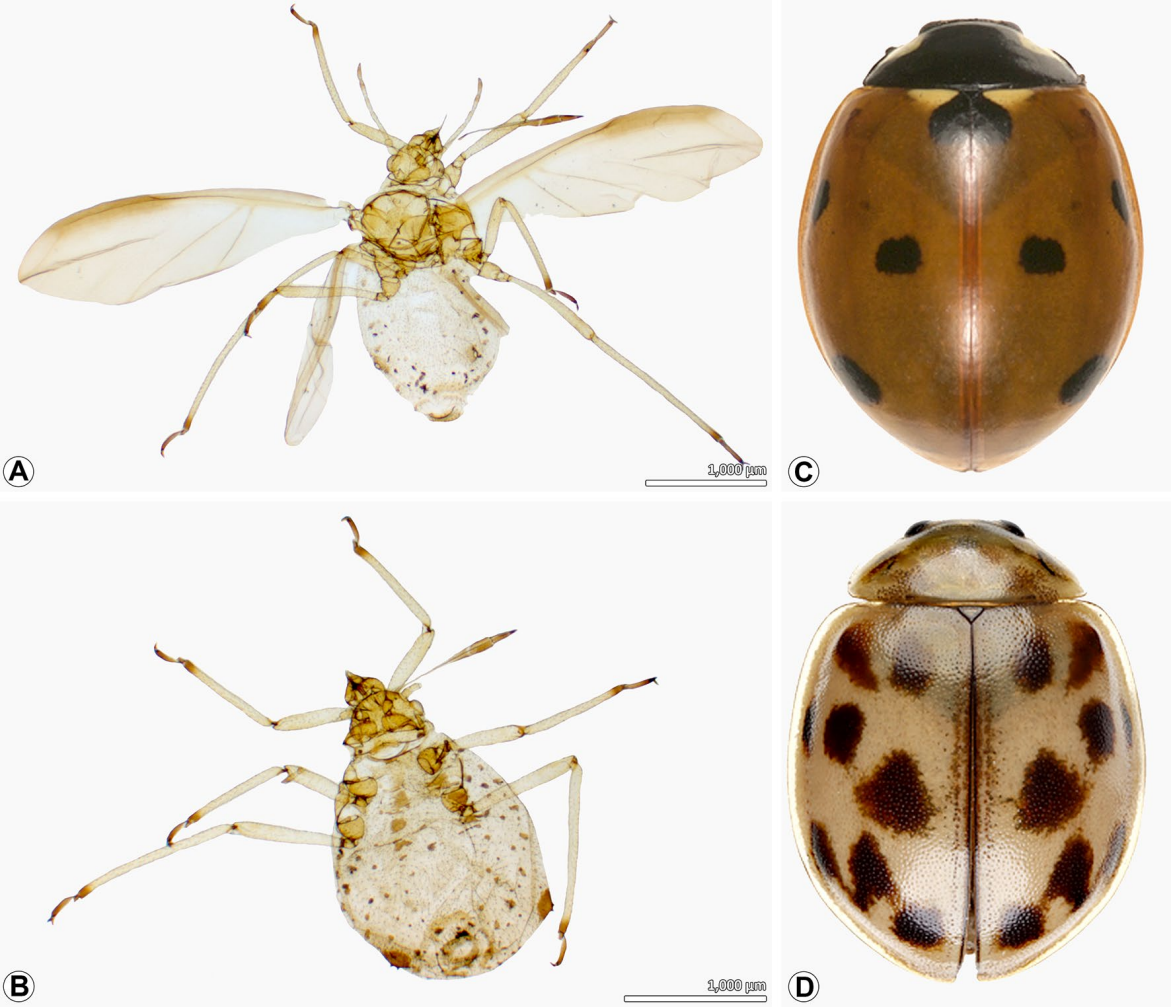
Dominant species associated with the Chinese thuja trees, *Platycladus orientalis* (L.) in three urban green spaces in Mashhad, Iran. (**A**) *Cinara tujafilina* Del Guercio, 1909, adult alate. (**B**) *Cinara tujafilina* Del Guercio, 1909, adult apterous. (**C**) *Coccinella septempunctata* Linnaeus, 1758. (**D**) *Oenopia conglobata* Linnaeus, 1758.

The Chinese thuja aphid, *Cinara tujafilina* Del Guercio (Hemiptera) was the dominant herbivore species across all three sites (Fig. 4A, B) with relative abundances (RA) of 86%, 93%, and 95% in Lashkar, Sadaf and Sajjad sites, respectively (Table 1). As for natural enemies, the seven-spot ladybird, *Coccinella septempunctata* L. (Coleoptera) was the dominant species (Fig. 4C) with RA of 35% and 49 in Sadaf and Lashkar sites, while the ladybird, *Oenopia conglubata* was the dominant species at Sajjad (Fig. 4D) with RA of 21% (Table 1).

### Insect diversity

The results of the nested ANOVA showed that there was a significant effect on the diversity of herbivores and natural enemies at each sampling site (P < 0.0001) (Fig. 3A, B; Table S1), while the effect of the sampling site on the overall diversity (H + P) was not significant (P = 0.82) (Fig. S2; Table S1).

The two-way ANOVA results indicated that the herbivore diversity was affected (statistically significant) by both the sampling site (F(2,29) = 7.32, P = 0.005) and the sampling season (F(3,29) = 6.81, P = 0.003) (Fig. 3A). However, the effect that the sampling site has on herbivore diversity was independent of the sampling season, meaning no interaction was found between these two factors (F(6,29) = 2.17, P = 0.95) (Fig. 3A, Fig. S2).

In respect of the natural enemies, not only diversity was not affected significantly by both the sampling site (F(2,21) = 0.28, P = 0.75) and the sampling season (F(3,21) = 1.79, P = 0.17) (Fig. 3B), but also the interaction between the sampling site and the sampling season was not significantly (F(6,21) = 0.50, P = 0.80), meaning that these two factors were independent of each other (Fig. 3B, Fig. S2). Lashkar and Sadaf sites presented the highest diversity both of herbivores and natural enemies across the four seasons (Fig. 3A, B, Fig. S2; Table S2).

### Insect abundance

Results of two-way ANOVA revealed that there was not a statistically significant interaction between the effects of the sampling site and the sampling season (F(6,24) = 1.25, P = 0.31) (Fig. 3C) on herbivore abundance. In other words, they did not significantly predict herbivore abundance. Simple main effect analyses showed that the sampling season has a statistically significant effect on herbivore abundance (F(3,24) = 6.31, P = 0.003) (Fig. 3C). On the contrary, the sampling site did not have a statistically significant effect on herbivore abundance (F(2,24) = 2.72, P = 0.08) (Fig. 3C).

As for natural enemies, the two-way ANOVA showed a statistically significant interaction between the effects of the sampling sites and the sampling season ((F(6,21) = 4.81, P = 0.002) (Fig. 3D) on natural enemy abundance. Simple main effect analyses showed that the sampling season (F(3,21) = 12.5, P = 0.002) and the sampling site (F(2,21) = 5.61, P = 0.01) also have a statistically significant effect on natural enemy abundance (Fig. 3D).

### Air pollutants

**Figure 5:**
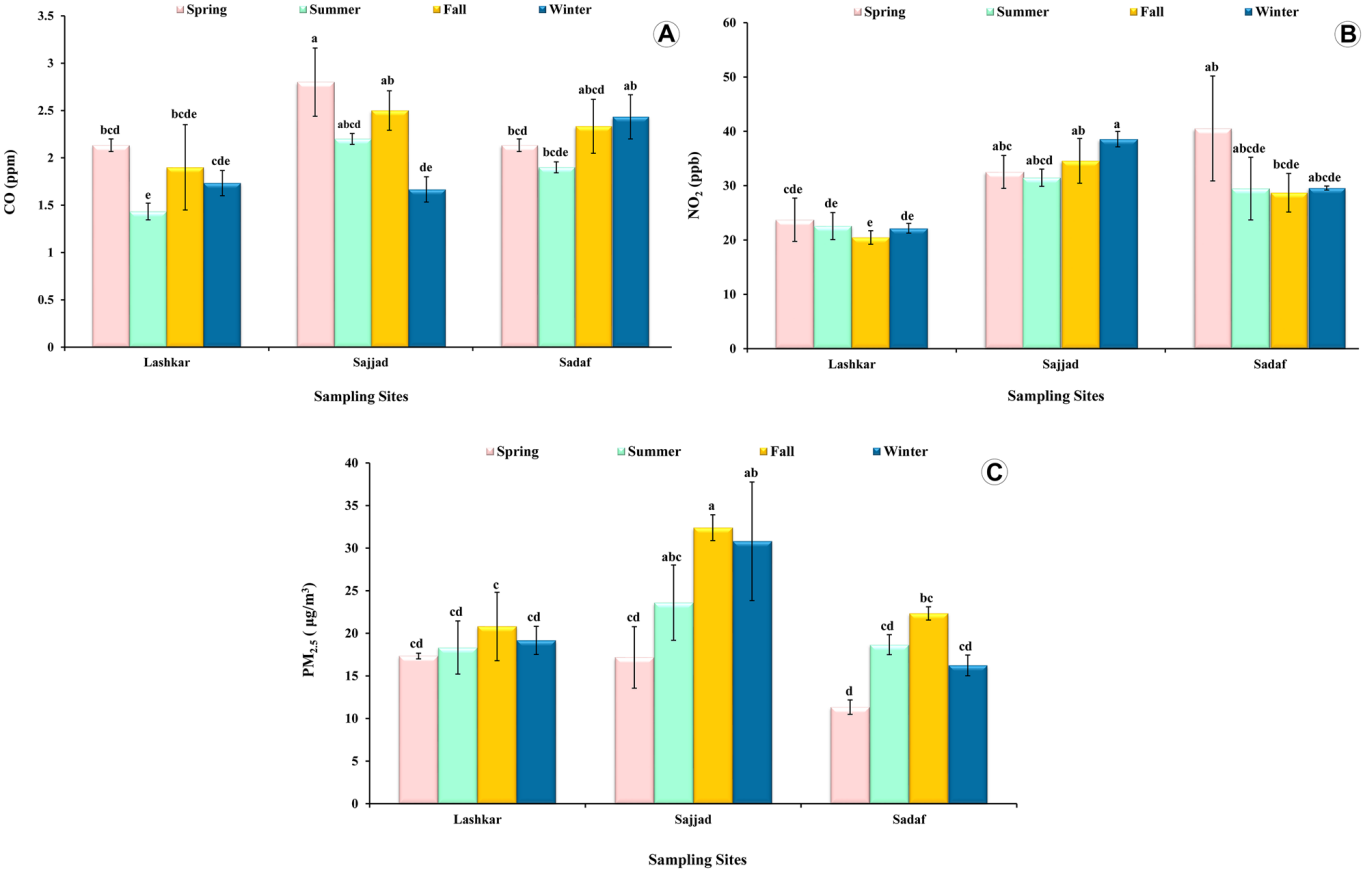
The mean concentration of (**A**) carbon monoxide, (**B**) nitrogen dioxide, (**C**) particles smaller than 2.5 microns, in three urban green spaces of Mashhad city from Iran. ± standard error, n = 3 for each pollutant at each site. Similar lowercase letters (e.g., bcd, abcd, bcde, abcde, etc.) indicate there is no significant difference among different seasons of each sampling site.

The CO concentrations varied significantly across the sampling sites (F(2,24) = 5.75, P = 0.009) and the sampling season (F(3,24) = 3.96, P = 0.026), but no interaction was detected between these two factors (F(6,24) = 2.10, P = 0.09) (Fig. 5A).

The NO_2_ concentrations varied among the sampling sites (F(2,24) = 13.8, P = 0.0001), but not among the sampling season (F (3,24) = 0.59, P = 0.627). Additionally, the interaction between the sampling site and the sampling season was not significant (F (6,24) = 0.72, P = 0.636) (Fig. 5B).

The PM_2.5_ concentrations differed significantly across the sampling sites (F(2,24) = 9.06, P = 0.001) and the sampling season (F (3,24) = 5.33, P = 0.006), but the effect of the sampling season was independent of the effect of the sampling site (F(6,241) = 1.09, P = 0.396) (Fig. 5C).

The highest concentration of CO was reported at the Sajjad site during the springtime (2.8 ppm) and the lowest at the Lashkar site in the summer (1.4 ppm) (Fig. 5A; Table S3). For NO_2_, the highest concentration was registered at the Sadaf site during the springtime (40.5 ppb) and the lowest at the Lashkar site in the fall (20.4 ppb) (Fig. 5B; Table S3). For PM_2.5_, the highest and lowest concentrations were observed at Sajjad and Sadaf but in different seasons. The highest (32.4 μg/m^3^) during the fall and the lowest (11.3 μg/m^3^) during spring (Fig. 5C; Table S3). The highest average concentration for each of the three pollutants was reported at the Sajjad site (Table S4). The lowest average concentration of CO and NO_2_ was reported at the Lashkar site and for PM_2.5_ was documented at the Sadaf site (Table S4).

### Association between air pollutants and insect diversity

**Figure 6:**
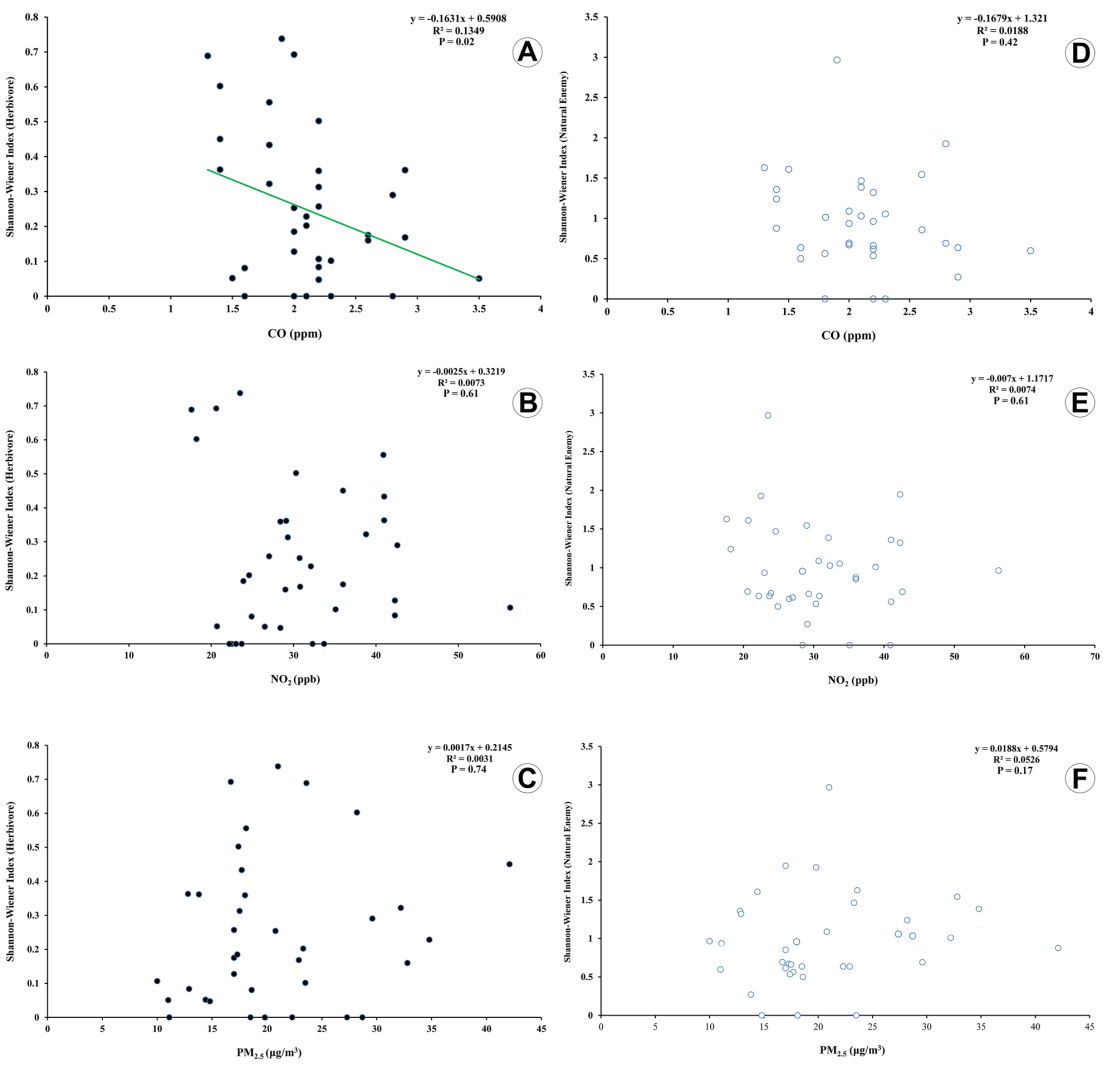
The Shannon–Wiener index regression of insect fauna associated with Chinese thuja trees*, Platycladus orientalis* (L.) with three air pollutant concentrations in urban green spaces of Mashhad city from Iran. Herbivores (**A**–**C**). Natural enemies (**D–F**). CO (**A, D**). NO_2_ (**B, E**). PM_2.5_ (**C, F**).

Herbivore diversity was significantly and negatively associated with CO concentration (F(1,34) = 5.30, R^2^ = 0.13, P = 0.02) (Fig. 6A), but no association was found neither with NO_2_ (F(1,34) = 0.25, R^2^ = 0.007, P = 0.61) nor with PM_2.5_ (F(1,34) = 0.11, R^2^ = 0.003, P = 0.74) (Fig. 6B, C). For its part, the association of natural enemy diversity with CO concentrations was not significant (F(1,34) = 0.65, R^2^ = 0.01, P = 0.42) (Fig. 6D), and similar to herbivore diversity no association was found neither with NO_2_ (F(1,33) = 0.25, R^2^ = 0.007, P = 0.61) nor with PM_2.5_ (F(1,34) = 1.89, R^2^ = 0.05, P = 0.17) (Fig. 6E, F).

### Association between air pollutants and insect abundance

**Figure 7:**
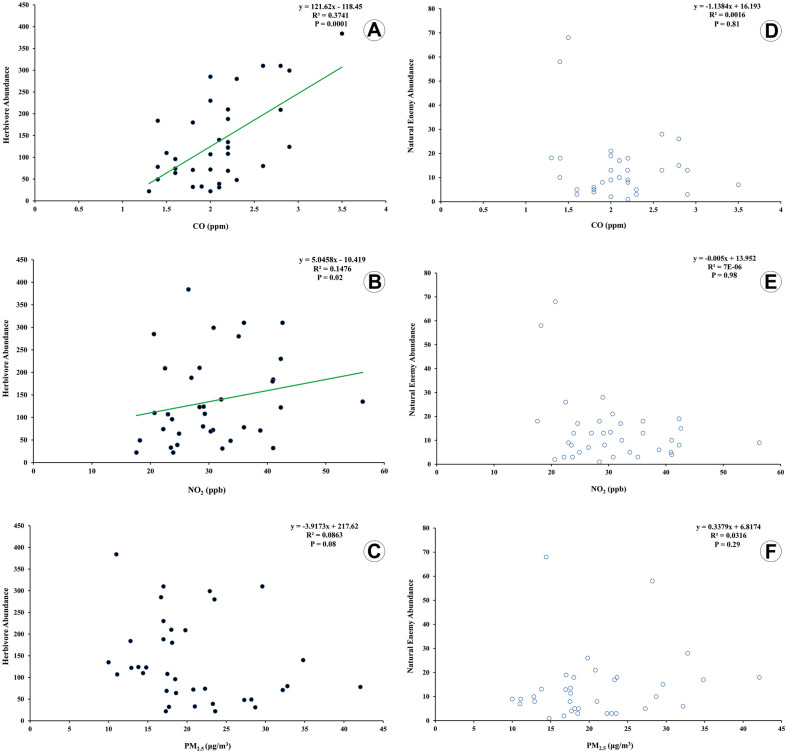
Abundance regression of insect fauna associated with Chinese thuja trees, *Platycladus orientalis* (L.) with three air pollutant concentrations in urban green spaces of Mashhad city from Iran. Herbivores (**A**–**C**). Natural enemies (**D**–**F**). CO (**A, D**). NO_2_ (**B, E**). PM_2.5_ (**C, F**).

The results of ANOVA showed that herbivore abundance was significantly and positively associated with the concentrations of CO (F(1,34) = 43.5, R^2^ = 0.56, P = 0.0001) and NO_2_ (F(1,33) = 5.89, R^2^ = 0.14, P = 0.02) (Fig. 7A, B), but not with PM_2.5_ (F(1,34) = 3.21, R^2^ = 0.08, P = 0.08) (Fig. 7C). Concerning natural enemy abundance, there was no a significantly associated with concentration levels of CO (F(1,34) = 0.06, R^2^ = 0.001, P = 0.81), nor NO_2_ (F(1,33) = 0.001, R^2^ = 0.0001, P = 0.98) or PM_2.5_ (F(1,34) = 1.11, R^2^ = 0.031, P = 0.29) (Fig. 7D–F).

### The status of air pollutants in Mashhad city

There are two types of particulate matter defined by their diameter for air quality regulatory purposes. PM_10_ (coarse) with a diameter of 10 microns or less and PM_2.5_ (fine) with an aerodynamic diameter less than or equal to 2.5 microns. Both types derive from different emissions sources and have different chemical compositions. PM_2.5_ pollution found in outdoor air is the result of emissions from the combustion of gasoline, oil, diesel fuel, or wood produce, as well as a significant proportion of PM_10_. PM_10_ also includes dust from construction sites, landfills and agriculture, wildfires and brush/waste burning, industrial sources, wind-blown dust from open lands, pollen, and fragments of bacteria^16^.

The PM_10_ standard threshold in Mashhad city was 150 μg/m^3^, which is higher than Australia, Canada, the EU, the USA, and the WHO standard thresholds. As for the PM_2.5_, Mashhad reported a value of 35 μg/m^3^, which is also higher than the WHO standard threshold. Mashhad’s NO_2_ standard threshold was 53 ppb, this average value is similar to Canadian and the USA thresholds but higher than Australian, the EU, and the WHO thresholds. As for the CO emission threshold for Mashhad, it was 9 ppm which is the threshold for Australia, the EU, the USA, and WHO, but less than the Canadian threshold. In the present study, the CO, NO_2_, and PM_2.5_ concentrations during all sampling seasons (2015–2016) and in all three urban green spaces sampled were lower than the Mashhad permissible threshold (Table 2).

**Table 2:** Standard levels of air pollutants given by American (USA), Australian, the European Union (EU), and the World Health Organization (WHO) including the permitted in Mashhad (Iran).

Pollutant	WHO	EU	Australia	USA	Canada	Mashhad
Ozone (8-h, ppb)	50	60	80	80	65	80
PM_10_ (24-h, μg/m^3^)	25	50	25	65	30	150
Sulphur dioxide (24-h, ppb)	8	48	80	140	115	140
Nitrogen dioxide (annual, ppb)	21	21	30	53	53	53
Carbon monoxide (8-h, ppm)	9	9	9	9	13	9
Lead (μg/m^3^)	–	0.5	0.5	1.5	–	–
PM_2.5_ (24-h, μg/m^3^)	25	–	–	–	–	35

*μg/m*^*3*^ micrograms per cubic meter, *ppb* part per billion, *ppm* part per million.

## Discussion

The Shannon–Wiener diversity index ranged between 1.5 and 3.5 and sometimes up to five. The index places a greater weight on species richness. High values indicate a community with higher species richness and as the distribution of individuals among the species becomes even (similarly abundant) there are no dominant species. This suggests a more complex food web and a more stable ecosystem.

The results here presented showed a significant and a negative association between herbivore diversity and CO concentration levels. However, no significant relationship was observed between levels of NO_2_ and PM_2.5_. On the contrary, associations between natural enemy diversity and the concentrations of all three air pollutants (CO, NO_2,_ and PM_2.5_) were not significant. However, the regression charts showed a slight increase in the natural enemy diversity index by increasing the concentration of PM_2.5_ levels and a slight decline with an increase in NO_2_ and CO concentration levels.

Following are some studies in which air pollutants exert a positive effect on the fauna of some insects. The growth rate of the rose aphid *Macrosiphum rosae* L. (Hemiptera: Aphididae) was higher in aphids grown in contaminated air than aphids grown in clear air^17^. Studies in laboratory conditions also have reported that at peak times, the acidic dust caused an increase both in nymph growth rate and population growth of the large pine aphid, *Cinara pinea*. Additionally, seasonality had a positive and significant effect both on the increase of pollutant concentrations and on the increase in population growth. Thus, in the first half of the summer, the population peaked under the influence of pollutants, but in the second half, there was no change in the pest population growth^18^. A direct correlation was also founded between the density and growth with different concentration levels of sulfur and nitrogenenic compounds, especially SO_2_. This was the case of two species of aphids (the waxy gray pine needle aphid, *Schizolachnus pienti* Davidson and the spruce shoot aphid, *Cinara pilicornis* Hartig) associated with *Pinus sylvestris* and *P*. *abies* (Pinaceae) in polluted areas^7^. Industrial pollution caused by a Ni–Cu smelting factory also has caused an increase in the diversity and abundance of ladybird species. Sampling sites near the factory showed greater diversity than those farther away, but it may have the opposite effect on other groups of natural enemies^19^. The species richness of ants and spiders increased with zinc concentrations in the air around a zinc smelting factory^20,21^.

On the other hand, other studies had reported the adverse effects of pollutants on insects. At very high levels air pollutants can sometimes cause a sudden decrease in the population of aphids^7^. The air contaminants such as SO_2_, NO_2_, and O_3_ decreased the *Abies fraseri* Poiret (Pinaceae) tolerance against the infestation of Adelgidae aphids (Hemiptera)^22^. The increase of both environmental stresses (temperature and water) and air pollutants (nitrogen, phosphorus, and lead) on the population of some insect pests had a negative effect on the natural enemies, but the severity of these changes depended on the type of environmental stress, species, and the insect growth stage^23^. Air pollutants influenced the richness of ladybirds (Coleoptera: Coccinellidae) associated with *Pinus sylvestris* forests around chemical factories, a gradual decrease was found over time, as well as a decrease in predatory coccinellid species richness in the forests near the factories^24^.

There are a considerable number of studies supporting and others contradicting the results reported here. Those discrepancies probably are related to sample size. Results here presented showed a positive and significant association between herbivore abundance and CO and NO_2_ concentration levels, while there was no significant correlation with PM_2.5_ concentration. Other studies had reported similar results. The population density of Mexican beetles, *Epilachna varivestis* Mulsant (Coleoptera: Coccinellidae) incremented as ozone concentrations increased^25^. This study found that high ozone concentrations reduced soybean resistance to Mexican beetle by increasing the feeding rate of adult insects and the growth rate of larvae^25^. Polluted air also caused an increase in the abundance and growth rate of Adelgidae aphids on *Abies fraseri* trees (Pinaceae, Fraser fir)^22^. Rising levels of CO_2_ also caused an increase in the amount of both glucose (47%) and secondary compounds (31%) in leaves of non-woody plants and also increased the prevalence of sucrose for insect pests^26^. The population of the pest *Melasoma lapponica* L. (Coleoptera: Chrysomelidae) was higher around a molten smelting factory than in clean air sites^27^. The prevalence of *Popillia japonica* Newman (Coleoptera: Scarabaeidae) and the ladybird, *Epilachna varivestis* Mulsant was positively correlated with the concentration of CO_2_, possibly due to an increase in the sugar content of soybean leaves in elevated levels of CO_2_^28^. A positive and significant correlation was founded between the soybean leaf pests with the O_3_ and CO_2_ levels in the air. As concentrations of these pollutants increased also there was an increment in the nutritional value of the leaves, the amount of damage, and pest population growth^29^. The increase in CO_2_ concentration caused a change in the nutritional value of white clover leaves, *Trifolium repens* L. (Fabaceae) for the western flower thrip, *Frankliniella occidentalis* (Pergande) (Thysanoptera: Thripidae). As a result, there was an increase in body size thrips, but its population size has not undergone any change^30^.

Air pollutants can also affect the behavior and function of natural enemies. However, in this study, the abundance and diversity of natural enemies were not significantly correlated with the levels of none of all three pollutants. Similar results have been previously reported. No significant correlation was found between the natural enemy abundance associated with the ash tree, *Fraxinus* L. (Oleaceae) with the concentrations of CO, NO_2,_ and PM_2.5_^19^. The SO_2_ concentrations were not significantly correlated with the species richness of grass flies (Diptera: Chloropidae) in two sampled contaminated areas^31^. An investigation of the effect of air pollutants on the natural enemies of the leaf beetle, *Melasoma lapponica* L. (Coleoptera: Chrysomelidae) near a molten smelting factory showed that predation of hoverfly larvae (Diptera: Syrphidae) and predatory bugs in the cleaned areas was approximately twice as much as the contaminated areas. Air pollution also increased the parasitism of leaf beetles by two flies, *Clenice nitidiuscula* (Zetterstedt) (Diptera: Tachinidae) and *Megaselia opacicornis* Schmitz (Diptera: Phoridae)^27^. Air pollutants, in addition to affecting the various biotic indexes of herbivore and predator insects, reduce the yield of the plants due to their reduced tolerance to the attack of insects, fungi, and other pathogens^6^.

### Conclusion and recommendations

Conifer trees are considered an important component in urban green spaces. They provide an effective way of reducing air pollution by absorbing carbon dioxide and converting it into oxygen and helping to improve air quality. Also, during photosynthesis, trees remove other chemicals, such as nitrogen oxides and ammonium produced in the air, sulfur dioxide, and ozone. Conifers have a widespread distribution and are attacked by a wide range of pests, including Curculionidae and Scolytidae (Coleoptera), sucker insects such as aphids (Hemiptera), and gnats in the family Sciaridae (Diptera) which are the vector of pathogenic fungi^32,33^. Therefore, it is very important to manage and maintain this ecological community with the development and expansion of urban green spaces.

In this regard, natural enemies reported here, such as ladybirds (Coleoptera), predatory bugs, and hymenopteran parasitoids such as *Encarsia formosa* Gahan (Aphelinidae) and *Pauesia hazratbalensis* Bhagat (Braconidae) would be used as potential biological control agents for integrated pest management, thus reducing the use of chemical pesticides and their harmful effects^34^. More studies must be done in open urban green spaces in Mashhad to know more about the insect fauna associated with these places. Likewise, it becomes necessary to know the native species and introduced ones. As well as evaluate the effects on the insect fauna that have different concentrations of these three air pollutants or other ones. Those data are paramount for an adequate implementation of integrated pest management programs. Insect fauna identification associated with other dominant trees in Mashhad urban green spaces such as *Platanus orientalis* L. (Proteales: Platanaceae, Oriental plane), *Robinia pseudoacacia* L. (Fabales: Fabaceae, Black locust), and *Pinus mugo* Turra (Pinales: Pinaceae, Mugo pine) may also contribute to the identification of biological control agents of many pests and subsequently, ensure that cities reduce the use of environmental toxins.

## Materials and methods

### Study area, pollutant data, and sampling design

**Figure 8:**
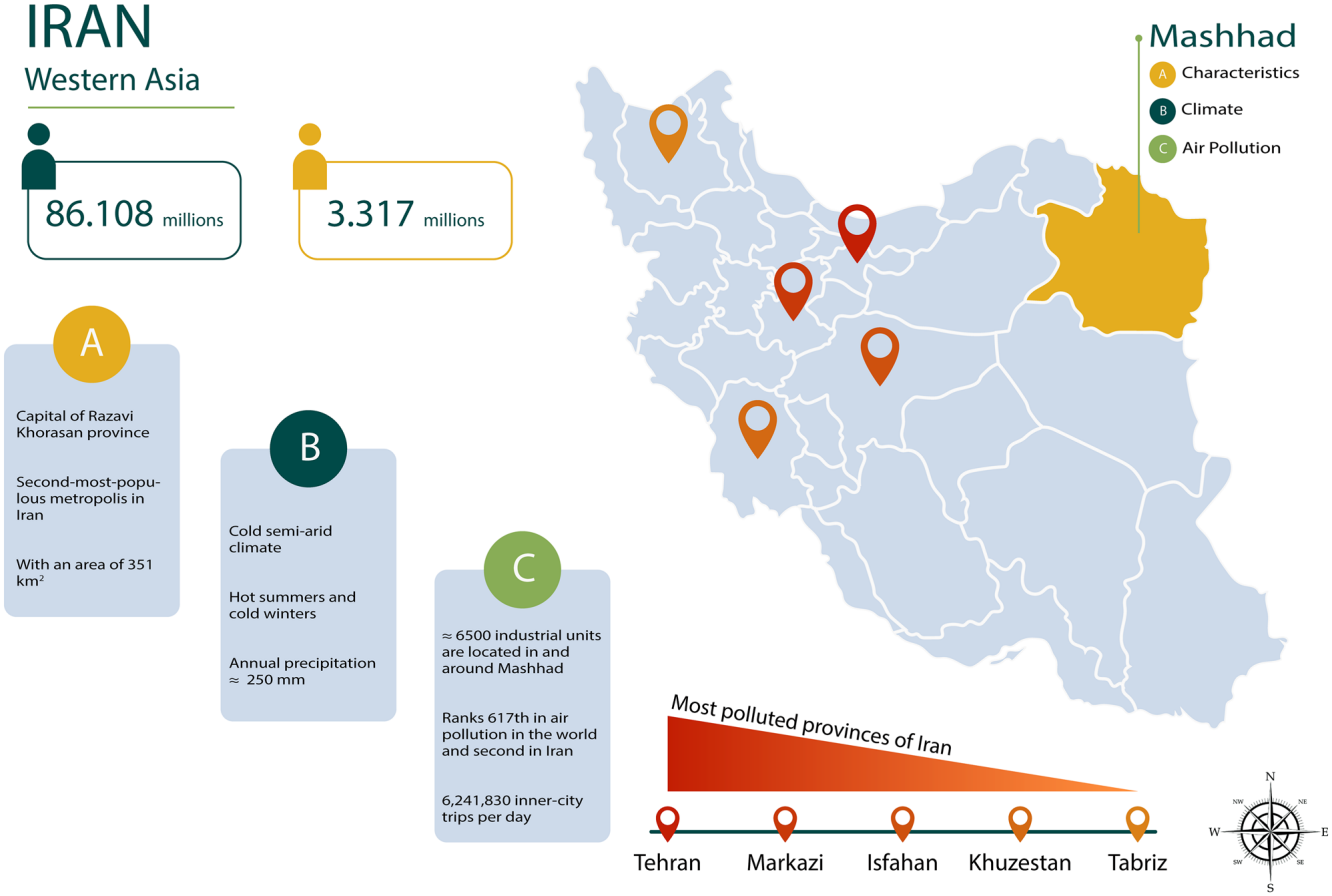
Map of Iran and study area in Mashhad along with features of city. The most polluted provinces of Iran are included on the map.

Mashhad is the second-most-populous metropolis in Iran with at least 3317 million people and the annual reception of more than 27 million domestic and foreign tourists (Fig. 8^35^). It is the capital of Razavi Khorasan province and is located in the northeast of the country. This city is located at 59° 2′ to 60° 37′ east longitude and 35° 43′ to 37° 7′ north latitude^35^. The city is characterized by a cold semi-arid climate with hot summers and cold winters^36^. The city of Mashhad is bounded on the south by the Binalood mountains, and on the north by the Hezar Masjed mountains. Its topographic slope is from southwest to northeast and the prevailing wind direction is northwest-southeast^35^. Mashhad has several tree-lined avenues with *Platycladus orientalis* as a dominant species which reaches a maximum height between ≈ 1.5 and ≈ 3 m and spreads ≈ 1.2 m along the avenues. In this study, trees of *P. orientalis* located on three sites from the northwest of the city were sampled, Lashkar (36° 20′ 52′′ N, 59° 27′ 34′′ E), Sadaf (36° 19′ 48′′ N, 59° 30′ 12′′ E), and Sajjad (36° 19′ 34′′ N, 59° 32′ 34′′ E), all places are at 995 m of elevation. The sites were chosen based on recommendations of the Mashhad Environmental Pollutants Monitoring Center (EPMC)^37^ including accessibility, the base of data available, and important and main sites, which have been collected from the annual report of air pollution monitoring sites.

Three air pollutants were evaluated, carbon monoxide (CO), nitrogen dioxide (NO_2_) whose concentrations are given in parts per billion (ppb) and parts per million (ppm) respectively, and particulate matter (PM) also called particle pollution with a concentration of air-born dust finer than 2.5 µm (PM_2.5_), concentration is given in micrograms per cubic meter (µg/m^3^). The air pollution data was compiled by the Mashhad Environmental Pollutants Monitoring Center^37^. We have chosen PM_2.5_ because the concentration of this pollutant in all districts of Mashhad was higher than the standard^37^. Hence, information from 2015 to 2016 for each site was facilitated by them.

Insect specimens were collected for a year, from March 27, 2015, to March 12, 2016. Collecting was carried out once a week (every Friday) for 2 h, from 10:00 am to midday. Three *P*. *orientalis* trees were randomly sampled at each site. For each tree, a total of 12 branches were sampled, and 20 cm from the apical part of each branch were covered with plastic bags and cut off. Bags with branches were transferred to the laboratory of the Department of Plant Protection, College of Agriculture, Ferdowsi University of Mashhad, Mashhad, Iran. In the laboratory, branches were examined and all insect specimens found were put into a 90% ethanol solution to preserve them. Specimens were first identified to order at the family level and following the taxonomic keys by Refs.^33,38,39^, and later specimens were sent to specialists who identified them at species (Table 1).

### Data analysis

We focused our attention on insects, and not on other organisms. According to their food habits, species were classified into two guilds, herbivores = (H: pests) and natural enemies = (P: predators + parasitoids). The relative abundances of each species were recorded, and the Shannon–Wiener index of species diversity was calculated to determine the dominant species of herbivores and natural enemies for each site.

The diversity of herbivores and natural enemies among sites was compared using a nested ANOVA (Analysis of variance). To assess if the air pollutant concentrations (CO, NO_2_, and PM_2.5_) had an effect on the insect fauna, a two-way ANOVA was done. Subsequently, the relationship between pollutants and insect abundance and between pollutants and insect diversity was estimated using linear regressions.

To investigate if the sampling site and the sampling season had an effect on the abundance of herbivores and natural enemies a two-way ANOVA was performed. Furthermore, it was evaluated whether or not there is an interaction effect between the sampling site and the sampling season on the abundance of herbivores and natural enemies.

A Tukey test was realized to figure out in which sampling sites the air pollutant concentrations differ. Additionally, this multiple comparison *post-hoc* test was also accomplished for comparing if the diversity of herbivores and natural enemies in each sampling site varies according to the sampling season.

Additionally, the permitted standard threshold of CO, NO_2_, and PM air pollutants in Mashhad city was compared to the announced indexes by American (USA), Australia, the European Union (EU), and the World Health Organization (WHO) standards.

All statistical analyses were conducted using the statistical software R ver. 4.2.0^40^, and the *nlme* (nested ANOVA), *RRPP* (ANOVA analysis and linear), *ggplot2* (generating the plots) packages, *aov* (two-way ANOVA), and *Tukey HSD* (post-hoc test) functions.

## Supplementary Information

Supplementary Information.

## Data Availability

The data that support the findings of this study are available from the corresponding author upon reasonable request.
