# Supplementary material for: Effects of carbon monoxide, nitrogen dioxide, and fine particulate matter on insect abundance and diversity in urban green spaces
Source: Sci Rep. 2022 Oct 25;12:17574. doi: 10.1038/s41598-022-20577-x (PMC9596448; doi:10.1038/s41598-022-20577-x)
Supplement: Supplementary file 1 — Supplementary Information. [file 41598_2022_20577_MOESM1_ESM.docx]

**Supplementary material**

**Title: Effects of carbon monoxide, nitrogen dioxide, and fine particulate matter on insect abundance and diversity in urban green spaces**

Authors: [Minoo Heidari Latibari](https://orcid.org/0000-0003-4158-8034), [Gholamhossein Moravvej](https://orcid.org/0000-0002-2432-7943), [Diana Carolina Arias-Penna](https://orcid.org/0000-0002-9601-3038) & [Mostafa Ghafouri Moghaddam](https://orcid.org/0000-0002-1942-9689)


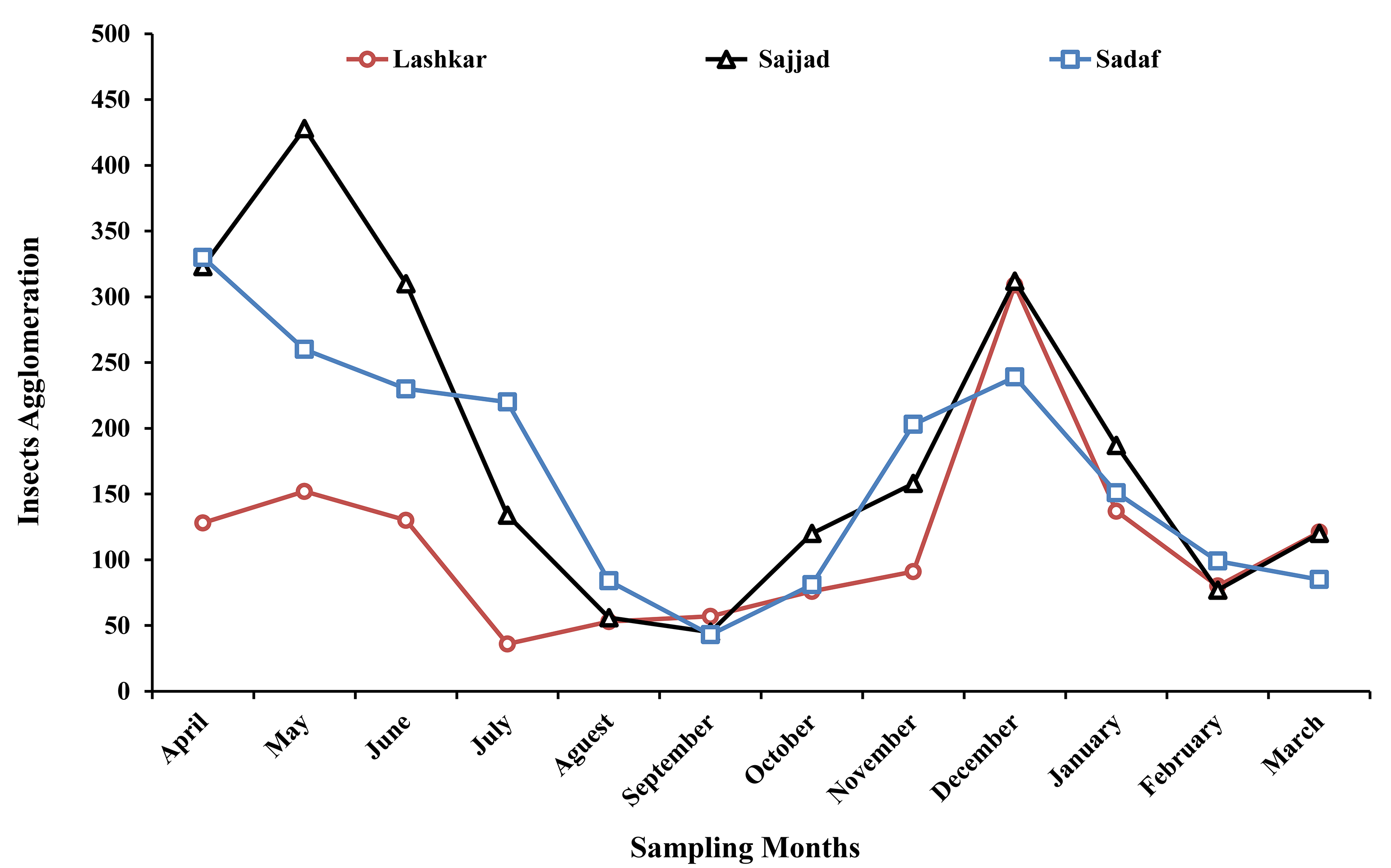


**Figure S1.** Abundance of insects associated with *Platycladus orientalis* (L.) in three urban green spaces in Mashhad city, Iran during March 2015-March 2016.


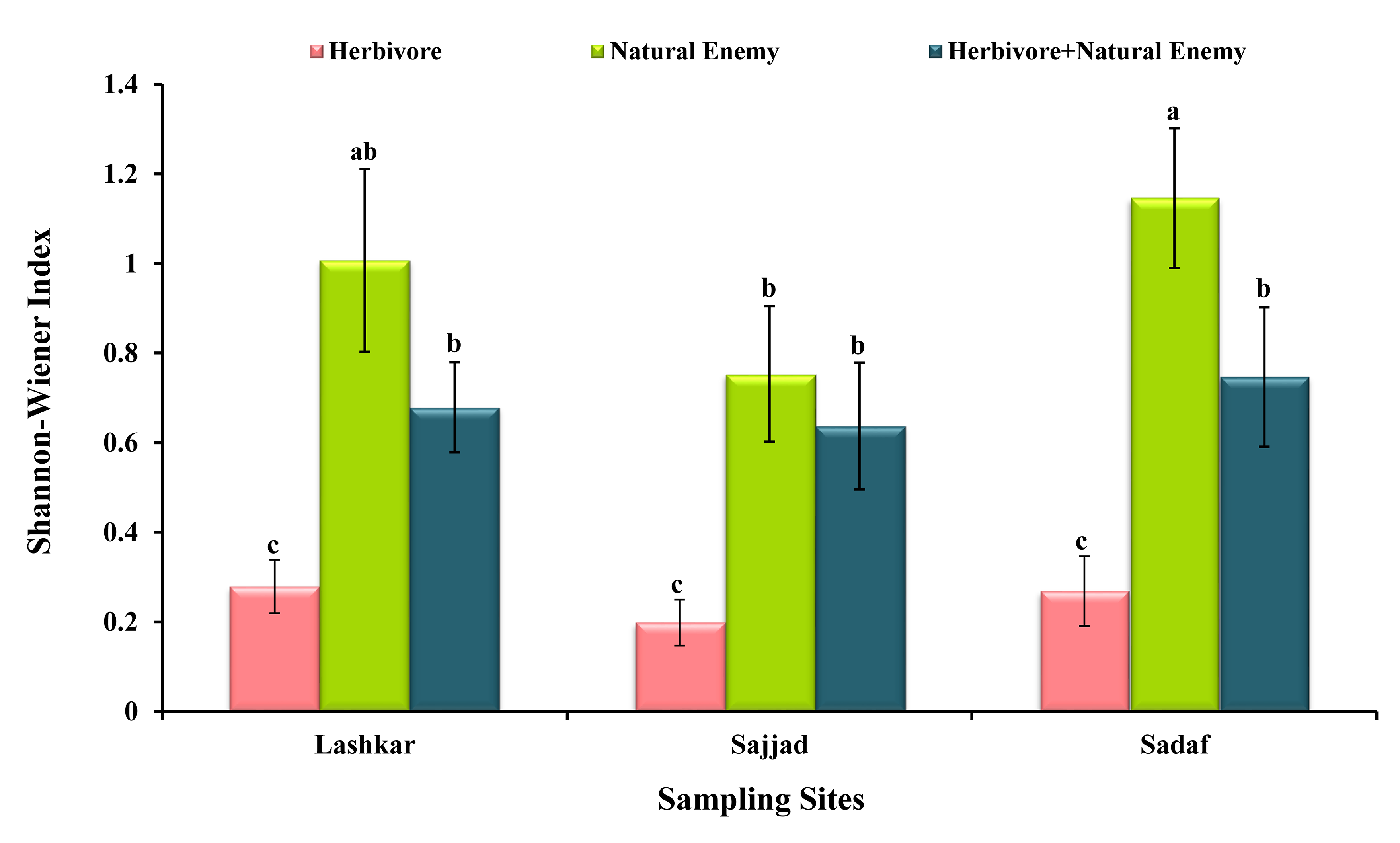


**Figure S2.** Shannon-Wiener index for insects, herbivores, and natural enemies associated with the Chinese thuja, *Platycladus orientalis* (L.) in three urban green spaces in Mashhad, Iran.

**Table S1.** Nested ANOVA and Shannon-Wiener index. Insects associated with *Platycladus* *orientalis* (L.) in three urban green spaces in Mashhad city based on different food habits and sampling sites from 2015 to 2016.

| Sources of changes | Df | F | P-value |
| --- | --- | --- | --- |
| Site | 3 | 0.20 | 0.82 |
| Insect food habits (Site) | 6 | 7.95 | <0.0001 |
| Error | 99 |  |  |
| Total | 108 |  |  |

**Table S2.** The maximum value of Shannon-Wiener index for multiple groups.

| Site | Herbivores | Natural enemies | Herbivores + Natural enemies |
| --- | --- | --- | --- |
| Lashkar | 0.73 | 2.96 | 1.37 |
| Sajjad | 0.55 | 1.54 | 1.75 |
| Sadaf | 0.69 | 1.94 | 1.80 |

**Table S3.** Comparison of air pollutants levels in the sampling sites with the approved indexes in Mashhad city.

| **Site** | **Season** | **CO (ppm)** | **NO_2_ (ppb)** | **PM_2.5_ (µg/m^3^)** |
| --- | --- | --- | --- | --- |
| **Lashkar** | **Spring** | 2.1 ± 0.06 | 23.7 ± 3.98 | 17.3 ± 0.33 |
|  | **Summer** | 1.4 ± 0.08 | 22.5 ± 2.48 | 18.3 ± 3.12 |
|  | **Fall** | 1.9 ± 0.45 | 20.4 ± 1.24 | 20.8 ± 4.01 |
|  | **Winter** | 1.7 ± 0.13 | 22.1 ± 0.89 | 19.1 ± 1.65 |
| **Sajjad** | **Spring** | 2.8 ± 0.36 | 32.5 ± 3.02 | 17.1 ± 3.60 |
|  | **Summer** | 2.2 ± 0.05 | 31.4 ± 1.58 | 23.6 ± 4.41 |
|  | **Fall** | 2.5 ± 0.20 | 34.5 ± 4.11 | 32.4 ± 1.51 |
|  | **Winter** | 1.6 ± 0.13 | 38.5 ± 1.41 | 38.8 ± 6.96 |
| **Sadaf** | **Spring** | 2.1 ± 0.06 | 40.5 ± 9.65 | 11.3 ± 0.84 |
|  | **Summer** | 1.9 ± 0.05 | 29.4 ± 5.76 | 18.6 ± 1.17 |
|  | **Fall** | 2.3 ± 0.28 | 28.7 ± 3.55 | 22.3 ± 0.77 |
|  | **Winter** | 2.4 ± 0.23 | 29.5 ± 0.03 | 16.2 ± 1.21 |

**Table S4.** The average concentration of air pollutants in the three urban green spaces sampled in Mashhad city, Iran. µg/m^3^ =micrograms per cubic meter, MO = carbon monoxide, NO_2_ = nitrogen dioxide, PM = particulate matter, ppb = parts per billion, ppm = parts per million.

| Site |  | Air pollutant |  |
| --- | --- | --- | --- |
|  | **CO** | **NO_2_** | **PM_2.5_** |
| Lashkar | 1.8 ± 0.18 ppm | 22.2 ± 2.15 μg/m^3^ | 18.9 ± 2.27 ppb |
| Sajjad | 2.2 ± 0.18 ppm | 34.2 ± 2.53 μg/m^3^ | 25.9 ± 4.12 ppb |
| Sadaf | 2.2 ± 0.16 ppm | 32.0 ± 4.83 μg/m^3^ | 17.1 ± 1.00 ppb |
